# Draft Genome Assembly of a *Wolbachia* Endosymbiont of *Plutella australiana*

**DOI:** 10.1128/genomeA.01134-17

**Published:** 2017-10-26

**Authors:** Christopher M. Ward, Simon W. Baxter

**Affiliations:** School of Biological Sciences, University of Adelaide, Adelaide, Australia

## Abstract

*Wolbachia* spp. are endosymbiotic bacteria that infect around 50% of arthropods and cause a broad range of effects, including manipulating host reproduction. Here, we present the annotated draft genome assembly of *Wolbachia* strain wAus, which infects *Plutella australiana*, a cryptic ally of the major *Brassica* pest *Plutella xylostella* (diamondback moth).

## GENOME ANNOUNCEMENT

*Plutella australiana* (Lepidoptera: Plutellidae) is endemic to Australia and morphologically cryptic with the worldwide *Brassica* pest, *Plutella xylostella* (1, 2). Despite the ability to hybridize under laboratory conditions, substantial variation across several traits has been documented between these two species, including prevalence of *Wolbachia* infection (3). *Wolbachia* spp. are a diverse group of intracellular bacteria that infect arthropods and nematodes and often act as reproductive parasites on hosts to promote their own transmission (4). Infecting mosquitos with specific *Wolbachia* strains has been used as a nontraditional method for blocking vector-borne diseases, demonstrating useful applications for these symbionts (5). In Australia, *Wolbachia* infection occurs in only 1.5% of *P. xylostella* moths, yet appears fixed among *P. australiana*. Whole-genome short-read sequencing of a whole *P. australiana* male moth facilitated the identification and genome assembly of a *Wolbachia* endosymbiont we named wAus.

*Plutella australiana* paired-end short reads (2 × 150 bp) were mapped to the wPip (GenBank accession no. NC_010981) and wMel (GenBank accession no. NC_002978) reference genomes using BWA-MEM (6) to separate putative *Wolbachia* reads from the host and other contaminants. The two resulting BAM files were converted to fastq using BEDTools (7) and concatenated, and duplicate sequences removed. This recovered 1,119,295 reads, of which 1,081,300 (96.61% of total reads) were properly paired (mapQ ≥ 5). The concatenated paired-end short reads were then assembled using Velvet version 1.2.10 (8), with a k-mer of 65. The wAus draft assembly has a total length of 1,158,805 bp across 95 contigs (*N*_50_ value, 19,935 bp), the largest of which is 72,415 bp. The NCBI Prokaryotic Genome Annotation Pipeline (https://www.ncbi.nlm.nih.gov/genome/annotation_prok/) identified annotations for 1,040 protein-coding genes, 43 pseudogenes, 34 tRNAs, 4 noncoding RNAs (ncRNAs), and 3 rRNAs (5S, 16S, and 23S). To test for non-*Wolbachia* bacterial sequence contamination in the assembly, contigs were divided into 1-kbp fragments and queried against a Kraken database (9) built from all complete bacterial references in RefSeq (https://www.ncbi.nlm.nih.gov/refseq/). Most fragments were classified as *Wolbachia* (98.7%), yet 1.3% reported no bacterial homology and were subsequently subjected to a BLAST search against the NCBI Genome database (https://www.ncbi.nlm.nih.gov/genome/) using Geneious version 10.1.3 (10). This failed to match known sequences, suggesting these regions may be specific to wAus.

Phylogenetic reconstruction using multilocus sequence typing genes (*coxA, gatB, ftsZ, fbpA*, and *hcpA*) (11) placed wAus into supergroup B, with 100% bootstrap support. Based on these five genes, wAus was most similar to the *Wolbachia* endosymbiont of *Culex quinquefasciatus* (wPip) and significantly different from other *Wolbachia* known to infect *Plutella* species (12). Recently, two genes causing cytoplasmic incompatibility in the *Wolbachia* strain wMel were identified as *cifA* (WD0631) and *cifB* (WD0632) (13); however, orthologs were absent from the wAus assembly and the *P. australiana* genome. Nevertheless, this draft genome provides an opportunity to investigate reproductive phenotypes associated with wAus infection, which may have future applications for biological control.

### Accession number(s).

This whole-genome shotgun project has been deposited at DDBJ/EMBL/GenBank under the accession no. MRWX00000000. The version described in this paper is the first version.
